# Shear and Compression Wrinkling Experimental Analysis with a Sandwich Beam Submitted to Three-Point Bending

**DOI:** 10.3390/ma18184286

**Published:** 2025-09-12

**Authors:** Befekadu Gashe, Abdulmaliq Alawode, Samuel Rivallant, Bruno Castanié

**Affiliations:** 1 Institut Clément Ader (ICA), CNRS UMR 5312, ISAE-SUPAERO, INSA, IMT Mines Albi, University of Toulouse, 31400 Toulouse, France; befekaduchalew@gmail.com (B.G.); alawodeabdulmaliq@gmail.com (A.A.); samuel.rivallant@isae-supaero.fr (S.R.); 2 Department of Structures for Engineering and Architecture, University of Naples Federico II, Via Claudio 21, 80125 Naples, Italy; 3 Department of Civil Construction, and Environmental Engineering, The University of Alabama, Tuscaloosa, AL 35487, USA

**Keywords:** three-point bending, sandwich, compression wrinkling, shear wrinkling, composite, finite element analysis

## Abstract

Wrinkling is a localized buckling phenomenon that significantly compromises the structural integrity of lightweight sandwich structures. The objective of this study was to validate the experimental design of a sandwich beam to observe the initiation of wrinkling under compression and, more specifically, under shear stresses. The specimen under consideration consists of glass fibre–epoxy skins with polymethacrylimide (PMI) ROHACELL^®^ foam cores. The experimental tests were monitored using Digital Image Correlation (DIC) techniques, in conjunction with displacement and force sensors. A linear buckling simulation was performed using Finite Element Analysis (FEA) in ABAQUS and was compared with both the experimental test results and analytical predictions. The simulations demonstrated a good correlation with both the experimental data and analytical models for compression wrinkling. In the case of shear wrinkling, the numerical analysis significantly overestimated the wrinkling load in comparison to the experimental results.

## 1. Introduction

A sandwich structure is a type of lightweight panel consisting of a core material sandwiched between two strong and stiff outer layers, known as skins, on the top and bottom [1]. The primary function of the lightweight core is to significantly enhance the sandwich’s bending stiffness and strength by being inserted between the face sheets with minimum increase in mass. These cores can be made from materials such as aluminum honeycomb or solid cores [2]. The utilization of composite laminates and metals in the fabrication of skins is a common practice. These structures are used in a variety of sectors, including automotive, shipbuilding [3], civil infrastructure [4], wind power generation, and aerospace [5].

**Figure 1:**
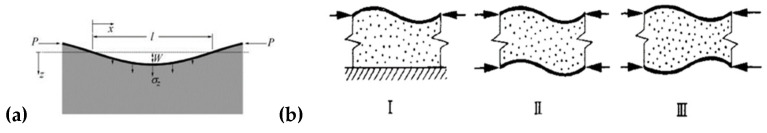
Basic wrinkling wave modelling (**a**) and wrinkling modes (**b**) [17].

Despite the numerous advantages of sandwich beams, they are susceptible to various potential failure modes that require careful consideration in design and usage, especially in aerospace applications [5,6]. The performance of sandwich structures is influenced by various elements, including geometrical dimensions, adhesive bonding between the skins and the core, and the stiffness and strength of the skins. Sandwich structures can display different failure modes when subjected to bending, shear, and in-plane loads. The initiation, propagation, and interaction of these failure modes are determined by the qualities of the constituent materials, the geometry, and the nature of loading [1,2,3,4,5,6]. These failure modes are interconnected, meaning that the worsening of an initial failure can trigger the development of other modes. The use of thin skins and lightweight cores in aeronautical structures makes them very sensitive to instabilities [5]. Two different scales of buckling modes can be observed in sandwich structures: global buckling, analogous to Euler’s buckling for columns, and wrinkling, which is a localized form of skin buckling [1,5,6,7,8]. To ensure the reliability and structural strength of sandwich structures, it is also crucial to understand and model how these different failure modes interact [9,10,11,12]. Most of the literature is dedicated to compression wrinkling of sandwich struts or panels [13,14,15,16]. Wrinkling in sandwich beams during compression or bending refers to a specific type of buckling when the compressed facing develops localized short-wavelength wrinkles, as illustrated in Figure 1. Wrinkling can be defined as the buckling of a compressed surface that is supported by a flexible material, known as the core. It is a common failure mode of sandwich beams, resulting in a diminution of beam stiffness and most often leading to a catastrophic failure. The wrinkling phenomenon is characterized by the interaction between the core and the facing of the sandwich structure. Therefore, the critical wrinkling load depends on the stiffnesses of the core and facing, the geometry of the structure, and the applied load [5,6].

Since Hoff and Mautner in 1945 [17], numerous authors have identified and studied the three wrinkling modes observed and illustrated in Figure 1b: skin wrinkling (I), antisymmetric wrinkling (II), and symmetric wrinkling (III) [5,6,7,8,9,10,11,12,13,14,15,16,17,18,19,20]. Many formulae have been developed, with varying degrees of efficiency. The primary formula employed in the field of aeronautics was developed using Hoff and Mautner and only depends on the material parameters of the facesheet and the core (Equation (1)).

(1) $$\sigma_{cr}=C\sqrt[{3}]{E_{c}E_{f}G_{c}}$$

where *E_f_* is the longitudinal Young’s modulus of the face, *E_c_* is the through-thickness Young’s modulus of the core, *G_c_* is the transverse shear modulus of the core, and *C* is a constant which is usually taken between 0.3 and 0.9 [1]. Hoff and Mautner [17] determined the constant *C* to be 0.91 based on the minimization of the total strain energy. This value of 0.91 was shown to be non-conservative when compared with experimental data and the effect of local imperfections [5,21]. A factor of 0.5 seems to be consistent as a knockdown factor for naval structures according to Fajerberg and Zenkert [21], whereas a factor of 0.33 is reported for aeronautics according to Castanié et al. [5].

Nevertheless, if much of the literature focuses on pure compression wrinkling, in actual structures, wrinkling may occur under combined loading [21,22,23,24], especially shear, which can also be regarded as tension/compression in the main directions at 45°. Fagerberg and Zenkerts [21] presented a method for estimating the wrinkling behavior of highly anisotropic sandwich panels under biaxial loading with experimental validation, unlike Birman et al. [25,26]. The authors identified a long wave perpendicular to the load direction in sandwich panels with substantial anisotropy under biaxial loading. This wave can be used as a tool to forecast the wrinkling behavior of the panels. The technique relies on the premise that wrinkling takes place at a certain angle, which is determined by computing the disparity between the applied tension and the maximum load that the panel can withstand without wrinkling. Khalili et al. [24] proposed an improved high-order theory for the modelling of wrinkling and developed an analytical solution for the static analysis of simply supported sandwich plates under biaxial in-plane compressive load.

Shear wrinkling can be a major concern for the sizing of civil engineering structures [4] and aeronautical structures [5]. However, most of the time, the authors focus on shear wrinkling of web cores [27,28] or honeycomb core [29,30,31] due to the out-of-plane shear forces of the sandwich under bending. For in-plane shear loads, according to Kassapoglou [32], in the field of aeronautics, a simple but conservative method consists of applying a compression formula in the compression direction to size the structure. Manshadi et al. [27,28] conducted an experimental investigation on a square sandwich panel consisting of E-glass fibre-reinforced polymer facesheets stabilized using a thick polyurethane (PU) foam core. The specimen was submitted to an in-plane biaxial compression–tension experiment using an Instron planar biaxial cruciform testing machine. Two typical shapes of wrinkling were observed on the facesheet: inward-oriented (local out-of-plane compression of the foam) or outward-oriented (local out-of-plane tension of the foam) wrinkles.

It would also be possible to analyze shear wrinkling by replacing laminate or metallic plates with sandwich plates in classical shear buckling tests. The first of these was proposed by Wagner [33] in the 1930s and allows post-buckling in shear of the web of an I-beam [34]. The second is a square deformable frame, initially proposed by the US Forest Laboratory for testing plywood [35,36], and classically used in the aeronautical field for analyzing post-buckling in shear of stiffened structures [37]. However, as can be seen from the references, these two test apparatuses are complex and expensive.

**Figure 2:**
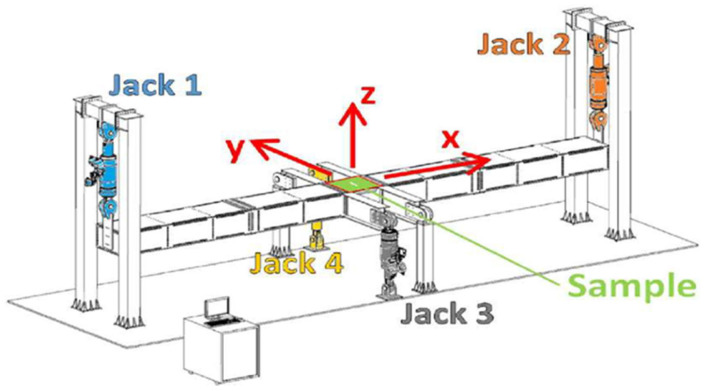
VERTEX test bench for combined loading [38,39].

**Figure 3:**
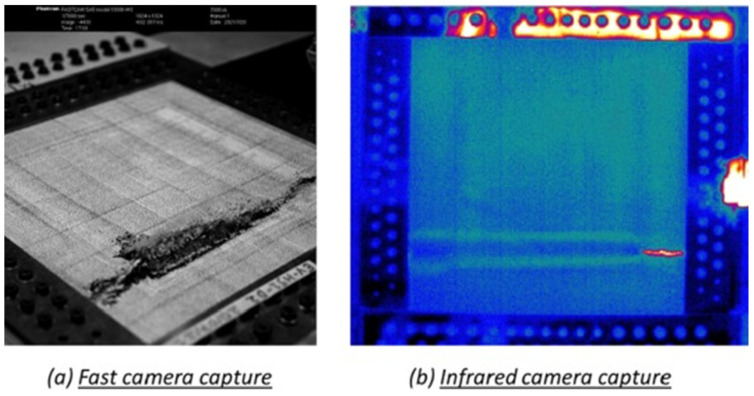
Wrinkling wave captured with a high-speed camera (**a**) and an infrared camera (**b**) [31]. The phenomenon is almost explosive.

Ginot et al. [38] investigated the wrinkling phenomenon in large-scale specimens. This approach aimed to provide more accurate insights into structural behavior, offering a more realistic alternative to previous small-scale coupon experiments. The VERTEX test bench, developed by Castanié et al. [39], is a testing bench under combined loading allowing for multiaxial stresses and replicating realistic boundary conditions, as shown in Figure 2. Five sandwich panel samples, consisting of PMI foam and Nomex^®^ honeycomb cores with carbon fibre-reinforced epoxy skins, close to the actual design of light aircraft, were tested. The experimental configuration comprised Stereo Digital Image Correlation (SDIC), infrared, and high-speed cameras to observe the deformation patterns and record the initiation of wrinkling, as illustrated in Figure 3. This study showed that the onset of wrinkling generally occurred at around 80% of the point at which the material failed, and the failure situations progressed swiftly. The occurrence of wrinkling was influenced by both the mechanical properties and geometric imperfections, especially at the boundaries between different core materials, resulting in the development of localized bending strains. This study further emphasized that Nomex^®^ honeycomb cores exhibited greater resistance to wrinkling than PMI foam cores due to their superior out-of-plane mechanical stiffness, despite having a lower density. The presence of minor imperfections, such as small indentations on the surface, has been demonstrated to have a considerable impact on the occurrence of wrinkling, highlighting the crucial role of precise manufacturing. This study highlights the importance of conducting extensive testing to gain a deeper understanding of the behavior of lightweight aircraft structures. Data obtained from such tests will be valuable in improving computational models, reducing the need for expensive physical testing, and enhancing the design and certification processes for lightweight aeronautical structures [38].

In summary, the literature shows that most research has focused on compression, with shear receiving very little attention. Furthermore, compression tests on sandwich specimens, which are difficult to perform, can also activate both symmetric and antisymmetric wrinkling modes, which are specific to the specimens themselves but not necessarily representative of real sandwich structures, which have a specific skin-wrinkling type mode depending on their geometry and materials—especially in the case of asymmetric sandwich structures [5].

Therefore, between large-scale specimens and tests, which are expensive and complex to handle, and simple beams under compression, which are not realistic enough, there is a need for an alternative, low-cost test. Therefore, this paper presents a specimen specially designed for analyzing skin buckling in shear or compression alone, based on a sandwich beam under three-point bending.

## 2. Materials and Methods

### 2.1. Materials and Manufacturing of Sandwich Beam Specimens

**Figure 4:**
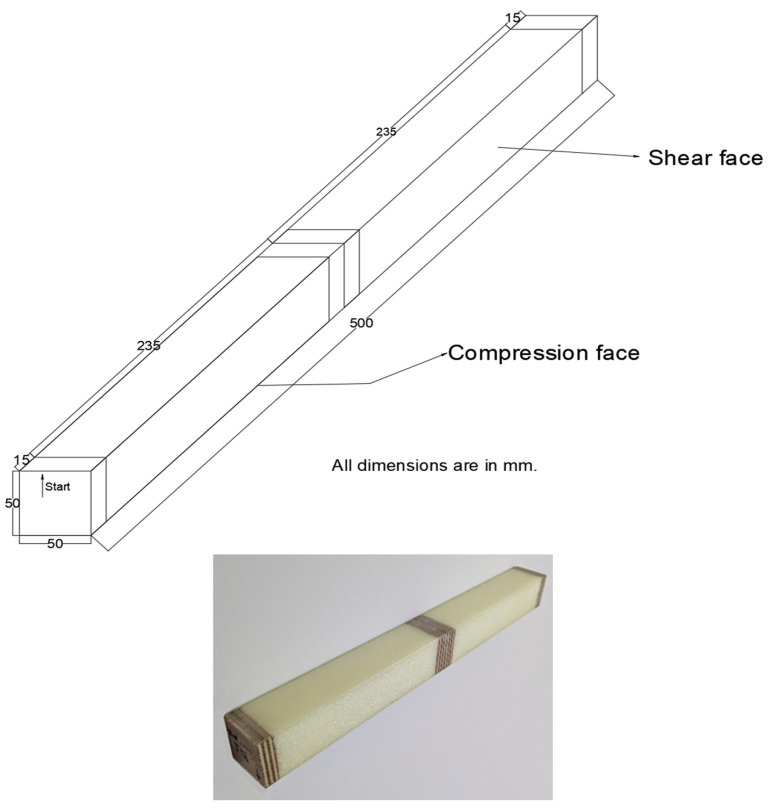
Specimen: overall geometry with dimensions (**upper**) and a picture (**lower**).

The specimen under consideration is a composite sandwich beam. Its dimensions are 50 mm in width, 50 mm in height, and 500 mm in length (see Figure 4). The composite skins of the sandwich beams were designed to achieve buckling under compression or shear. They are made of a combination of glass fibre–epoxy unidirectional layers and glass fibre–epoxy fabric layers. The specimen’s core is made of isotropic PMI ROHACELL^®^ 51 WF foam (Evonik Industries, Darmstadt, Germany). Table 1 presents the mechanical properties of the skin materials, Table 2 describes the mechanical properties of the isotropic foam core (both obtained from datasheets), and Table 3 displays the mechanical characteristics of the plywood material used at the loading and support locations in the sandwich structure to prevent local crushing of the foam. These plywood supports are made using blocks measuring 50 × 50 × 15 mm^3^, as shown in Figure 4. There are four blocks in total: one at each edge and two in the centre. The plywood is stacked with seven plies: four Okoumé plies (1.5 mm thick) starting at the surface, and three birch plies (3 mm thick).

**Figure 5:**
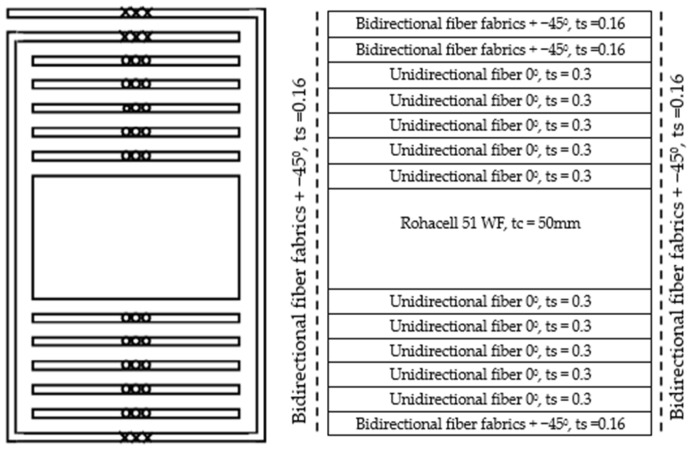
Stacking sequence to achieve shear wrinkling on the side faces.

In order to induce shear wrinkling, the specimens were designed with relatively thick skins in the upper and lower skins, which will prevent local compressive buckling, while a very thin side face with only one ply at 45° is designed. Like the foam core of the sandwich, this very thin face is subjected to shear, and wrinkling is expected to occur (Figure 5). Five unidirectional plies were stacked at 0° on the top and bottom of the core, and a 45° bidirectional fabric ply was added to cover all faces of the specimen, with an overlap at the top. Hence, the different lay-ups are as follows:

- Top: five 0° UD plies + two 45° fabric plies = [0_5_/(+45/−45)_2_];
- Bottom: five 0° UD plies + one 45° fabric ply = [0_5_/±45];
- Side faces: one 45° fabric ply = [±45].

**Figure 6:**
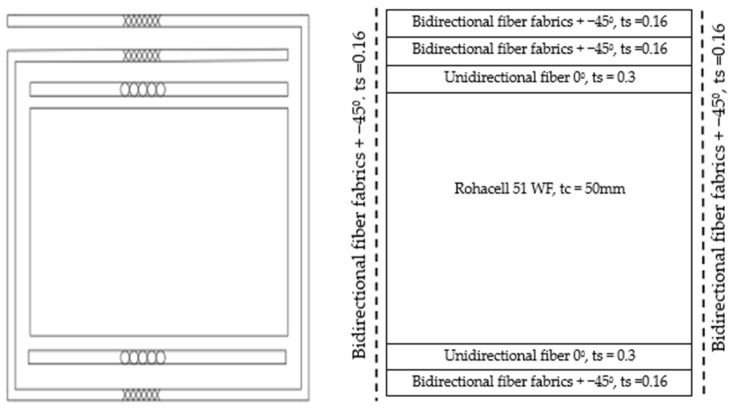
Stacking sequence to achieve compression wrinkling on the lower skin.

To induce compression wrinkling, a second type of stacking was defined (Figure 6). These specimens were designed with a thin lower skin where compression is induced by bending. This very thin skin will be prone to wrinkling. Only one unidirectional ply was stacked at 0° on the top and bottom of the core, and a second 45° bidirectional fabric ply was added to cover all faces of the specimen, with an overlap at the top. Therefore, the thickness on the side will be sufficient to delay shear buckling. Hence, the different lay-ups are as follows:

- Top: one 0° UD ply + two 45° fabric plies = [0/(+45/−45)_2_];
- Bottom: one 0° UD ply + one 45° fabric ply = [0/±45];
- Side faces: one 45° fabric ply = [±45].

The production of the specimens was accomplished by means of a hand lay-up technique with manual impregnation. Initially, non-crimp dry E-glass UD and fabrics were cut to the appropriate dimensions. Furthermore, the PMI ROHACELL^®^ 51 WF foam core was meticulously cut in accordance with the specified geometry, ensuring the prevention of initial dents in the foam that could lead to undesired failure modes. The wooden components were affixed to the cores using adhesive. Then composite plies were arranged in accordance with the sequences that had been previously presented. For the resin, a combination of Araldite LY 5052 and its corresponding hardener, Aradur 5052, both manufactured by HUNTSMAN (Everberg, Belgium), was employed with a 50:19 weight ratio. Subsequently, the specimen was placed within a mould to ensure a flat surface on all four faces and to impose pressure during the curing process.

### 2.2. Three-Point Bending Tests

**Figure 7:**
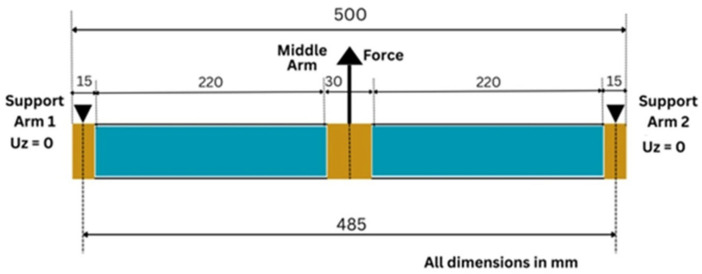
Three-point bending test principle (blue: foam; brown: plywood).

The sandwich specimen was positioned on the foundation of a manually operated three-point bending machine. As illustrated in Figure 7, the two outer arms functioned as supports, while the central arm was utilized for the application of the load. In order to circumvent the risk of localized failure resulting from crushing of the foam core during the application of the load, wooden components are inserted into the core during manufacturing in front of each arm.

**Figure 8:**
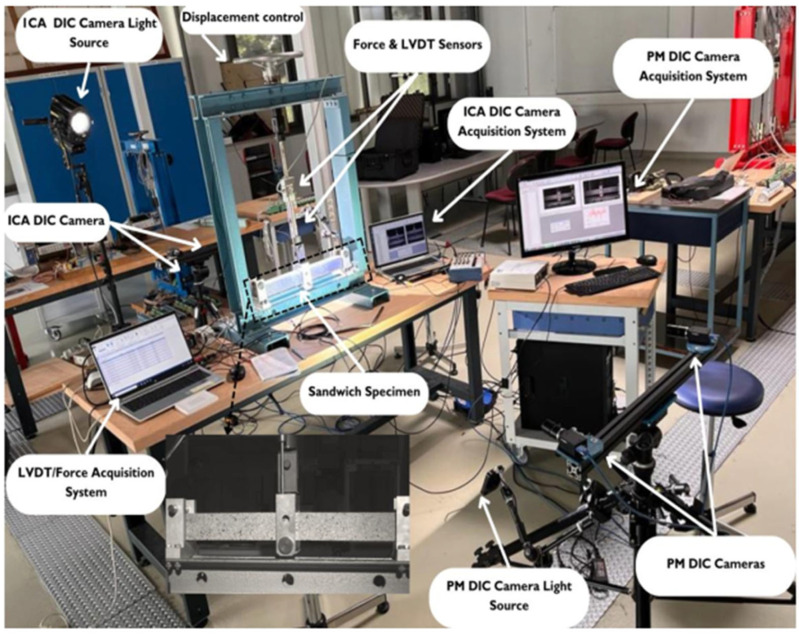
Experimental setup for the three-point bending test in the shear wrinkling configuration.

A 25 mm LVDT displacement sensor (HBK France, Sucy-en-Brie, France) was mounted on the middle arm to measure the vertical displacement during loading for the shear wrinkling specimen, and a 20 mm LVDT displacement sensor (HBK France, Sucy-en-Brie, France) was used for the compression wrinkling specimen, as illustrated in Figure 8. In addition, for both shear and compression specimens, an HBM S9M/10kN force sensor (HBM France, Sucy-en-Brie, France) was attached to the middle arm to measure the force being exerted. The LVDT displacement sensor and the force sensor were connected to an acquisition system for the purpose of data storage. Two DIC cameras with a resolution of 5 megapixels (2462 × 2052, IDS, Obersulm, Germany) were also used to observe the deformation of the specimen and the expected wrinkling. The cameras were positioned on the sides of the specimen to observe shear failure in the side faces, while in the other test setup, the cameras were placed at the bottom to visualise the lower skin of the specimen. Therefore, it was necessary to elevate the supports in the second configuration test (not represented in Figure 8).

## 3. Experimental Results

### 3.1. Experimental Results of Compression Wrinkling Tests

**Figure 9:**
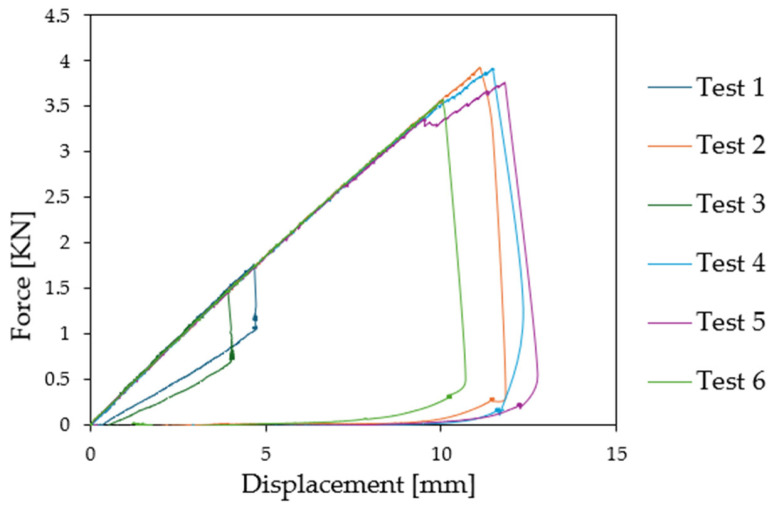
Force [kN] as a function of relative displacement [mm].

The Digital Image Correlation (DIC) cameras were first employed to document the initial imperfections present on the surface of the lower skin of the sandwich specimens prior to the application of any external force, since this point can influence the wrinkling load. The initial maximum deflection was found to be approximately 0.1 mm for the six specimens. Subsequently, during the loading process, force and displacement were recorded, and the force was plotted as a function of displacement for the six compression test specimens (see Figure 9). As demonstrated in Figure 9, all specimens show identical linear properties at the onset of the loading. However, a discernible discrepancy is evident in the force exerted at buckling initiation. Specifically, four of the specimens exhibited the first indications of wrinkling (load drop) when subjected to high loading conditions (Test 2: 3.91 kN, Test 4: 3.91 kN, Test 5: 3.95 kN, and Test 6: 3.56 kN), while on the other hand, two specimens (Test 1 and Test 3) failed at lower force values (1.46 kN and 1.79 kN, respectively). Nevertheless, this level of dispersion is not unusual for local buckling.

**Figure 10:**
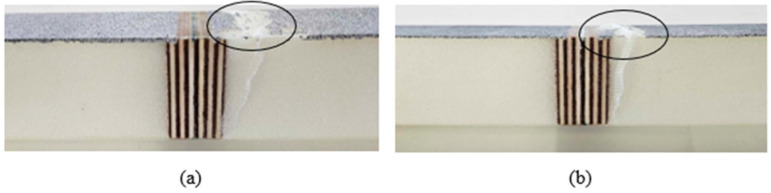
Wrinkling in compression, leading to a hollow (**a**) or a bump (**b**) on the bottom skin.

With regard to the fracture of the specimens, failure was invariably located beneath the foam core, in close proximity to the central wooden piece, and induced by wrinkling. This outcome was anticipated, given that this area exhibits the highest levels of compressive stress within the skin. However, two distinct shapes were observed, the wrinkling resulting either in a hollow or in a bump on the surface of the beam. These two results are illustrated in Figure 10, with the surface showing the DIC speckle pattern being the bottom skin in compression during the test.

### 3.2. Experimental Results of Shear Wrinkling Tests

**Figure 11:**
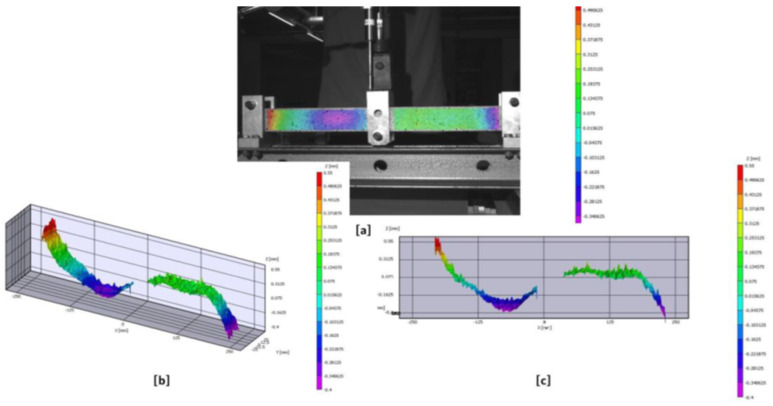
Test-A1 Digital Image Correlation (DIC) initial out of plane displacement result shown in (**a**) 2D-plot view; (**b**) 3D-plot view; and (**c**) XZ-plane plot.

Six specimens were also tested to get shear wrinkling. As in the compression wrinkling tests, Digital Image Correlation (DIC) cameras were employed to record the initial imperfections of the side faces of the sandwich specimens subjected to shear wrinkling before the application of any external force. As illustrated in Figure 11, this out-of-plane (z) imperfection is evident on one side of test specimen A1, with a discernible imperfection present in the purple zone. The level of imperfection measured (shape, size, and depth) varies from one sample to another, reaching a maximum of 0.8 mm in depth magnitude on certain specimens. This variability can be attributed to the manual lay-up production method used during manufacturing.

**Figure 12:**
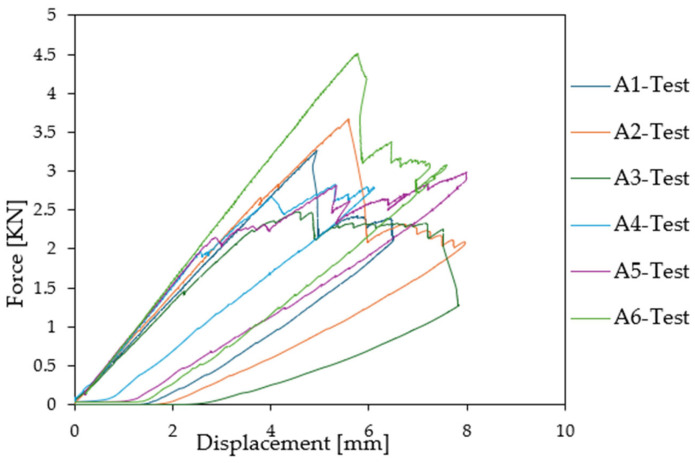
Force [kN] as a function of relative displacement [mm] for the shear experimental test.

Then an increasing load was applied to the beam to reach wrinkling. The force was plotted as a function of displacement for the six compression test specimens. It is important to observe that all specimens manifest equivalent linear properties at the initiation of loading procedure, as demonstrated in Figure 12. However, a discernible discrepancy in the force exerted at the initiation of buckling is also observed here. Specifically, three of the specimens showed the first wrinkling when subjected to high loads (Test A1: 3.25 kN, Test A2: 2.65 kN, and Test A6: 4.39 kN) while for the three other specimens (Test A3, Test A4, and Test A5) wrinkling appeared at lower values (1.438 kN, 1.486 kN, and 1.487 kN, respectively).

**Figure 13:**
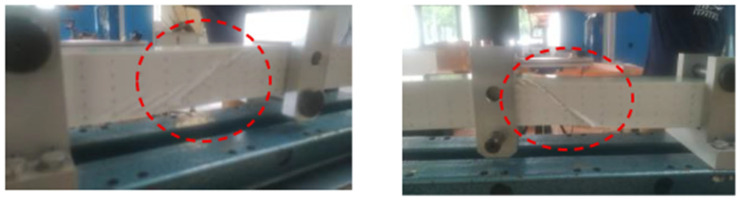
Two typical shear wrinkling patterns.

Wrinkling was observed in all specimens, displaying the characteristic 45° wrinkle patterns shown in Figure 13. The first wrinkle occurred randomly on one of the four possible faces (front, rear, left, or right of the central wooden piece). Furthermore, there is also a discrepancy regarding the location of the initial wrinkle within each face. In the left image of Figure 3, the wrinkle is located at the centre of the face; in the right image, it is positioned close to the central wooden piece. These discrepancies are attributable to local imperfections or a different failure scenario, such as failure of the core in tension or compression, or failure of the interface. As with compression wrinkling tests, wrinkling can result in either a hollow or a bump. However, a significant difference compared to the compression case is the manner in which new wrinkles emerge during continued loading. These new wrinkles appear around the initial one on the same face, as shown in the left-hand picture in Figure 13, and also on the other faces.

Nevertheless, the proposed experimental technique has demonstrated its effectiveness in creating shear wrinkling despite its intrinsic complexity.

## 4. Finite Element Modelling of the Tests

### 4.1. Description of the Finite Element Model

**Figure 14:**
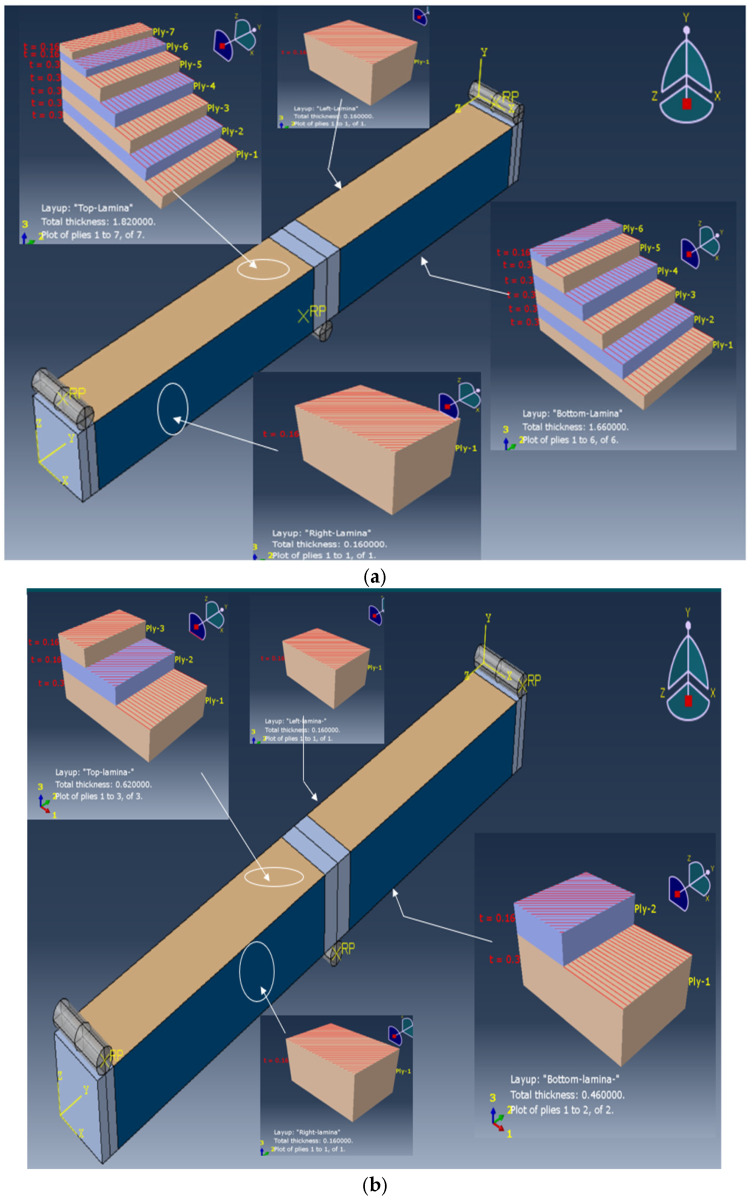
Finite elements models with face lay-up definition: shear wrinkling (**a**) and compression wrinkling (**b**).

The Abaqus Standard Version 2022 was used. The numerical computation was performed on an Intel Core i9 computer (Dell France, Montpellier, France). A full-scale model was developed, and R3D4 rigid body elements were selected to represent the boundary conditions for both the two extremity supports and the loading arm (see Figure 14). Conventional shell elements (S4R) were used for the outer face skin material, while the core material and plywood were modelled using C3D8R solid elements with reduced integration.

### 4.2. Mesh Convergence Study for Compression Wrinkling

**Figure 15:**
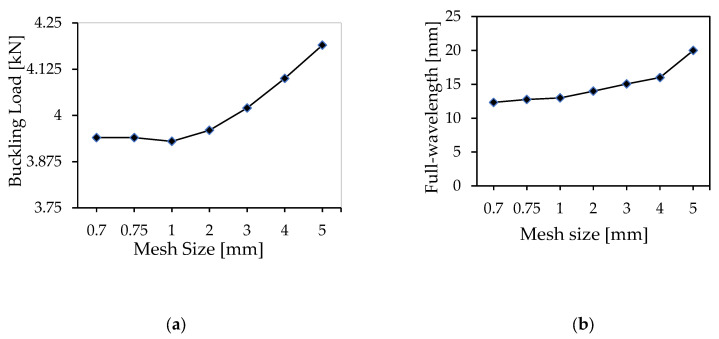
Mesh convergence study for the compression wrinkling case: critical load (**a**) and full wavelength (**b**).

A mesh convergence study was conducted for the compression wrinkling mode to examine the impact of mesh size on the buckling load eigenvalue and wrinkling pattern for wavelength evolution. Seven different mesh sizes ranging from 0.7 mm to 5 mm were tested, including 0.75 mm, 1 mm, 2 mm, 3 mm, and 4 mm. The buckling load eigenvalue in the compression numerical simulation was found to be 3.94 kN for the smallest mesh size of 0.7 mm, and 4.20 kN for the largest size of 5 mm (see Figure 15a). As can be seen in the figure, there is a steady and proportional increase in the buckling load with an increase in mesh size. Notably, there is convergence in the buckling load values when the mesh sizes range from 1 mm to 0.7 mm.

The 0.7 mm mesh size had a measured full wavelength of 12.73 mm, while the 5 mm mesh size had a measured full wavelength of 20 mm, as shown in Figure 15b. Intermediate mesh sizes produced wavelengths of 12.76 mm (0.75 mm mesh), 13 mm (1 mm mesh), 14 mm (2 mm mesh), 15.06 mm (3 mm mesh), and 16 mm (4 mm mesh). The graph shows a consistent, proportional increase in full wavelength as mesh size increases. Notably, there is a convergence in full wavelength for mesh sizes ranging from 1 mm to 0.7 mm.

**Figure 16:**
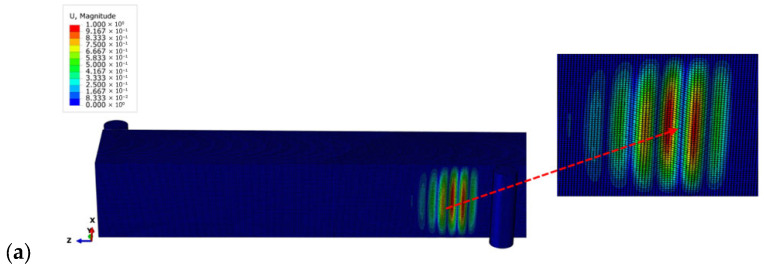

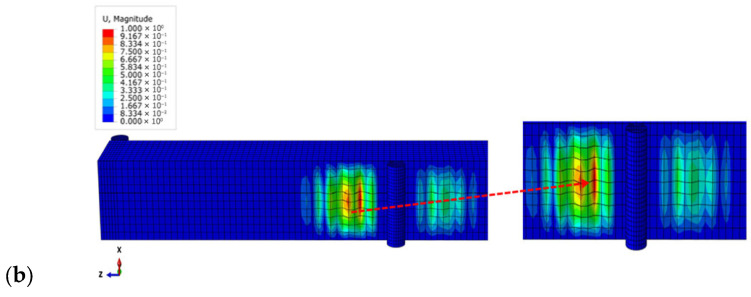
Wrinkling pattern for the compression wrinkling case: 0.70 mm mesh size (**a**) and 5 mm mesh size (**b**).

Figure 16 shows the results of the numerical simulation study for the minimum and maximum mesh sizes, focusing on the shape of the wrinkles. These figures show how the pattern of wrinkling changes as the mesh sizes vary. Wrinkling occurs on the bottom compression face of the sandwich panel and, as in the experimental results, is located near the central support.

### 4.3. Mesh Convergence Study for Shear Wrinkling

**Figure 17:**
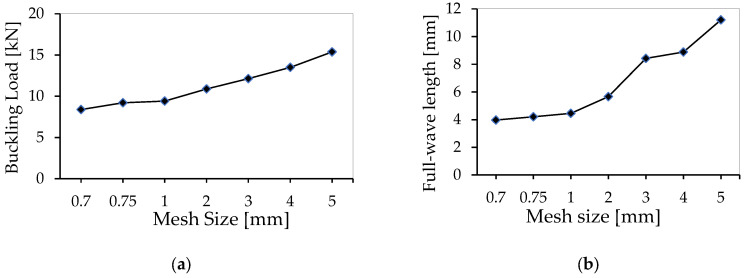
Mesh convergence study for the shear wrinkling case: critical load (**a**) and full wavelength (**b**).

A mesh convergence study was also conducted for the shear wrinkling failure test to examine the impact of mesh size on the eigenvalue of the buckling load, as well as the wrinkling pattern and wavelength evolution. The same seven mesh sizes were tested, ranging from a minimum of 0.7 mm to a maximum of 5 mm, including 0.75 mm, 1 mm, 2 mm, 3 mm, and 4 mm. The buckling load eigenvalue in the shear numerical simulation was found to be 8.38 kN for the smallest mesh size of 0.7 mm and 15.78 kN for the largest mesh size of 5 mm, as shown in Figure 17a. As can be seen in the figure, there is a steady and proportional increase in the buckling load with an increase in mesh size. Notably, convergence in the buckling load values occurs when the mesh sizes range from 1 mm to 0.7 mm.

The 0.7 mm mesh size had a measured full wavelength of 3.97 mm, while the 5 mm mesh size had a measured full wavelength of 11.2 mm (see Figure 17b). Intermediate mesh sizes produced wavelengths of 4.2 mm (0.75 mm mesh), 4.45 mm (1 mm mesh), 5.66 mm (2 mm mesh), 8.42 mm (3 mm mesh), and 8.88 mm (4 mm mesh). The graph shows a consistent, proportional increase in full wavelength as the mesh size increases. Notably, there is convergence in the full range of wavelengths for mesh sizes from 1 mm to 0.7 mm. However, shear wrinkling is much more sensitive to mesh size than compression wrinkling.

**Figure 18:**
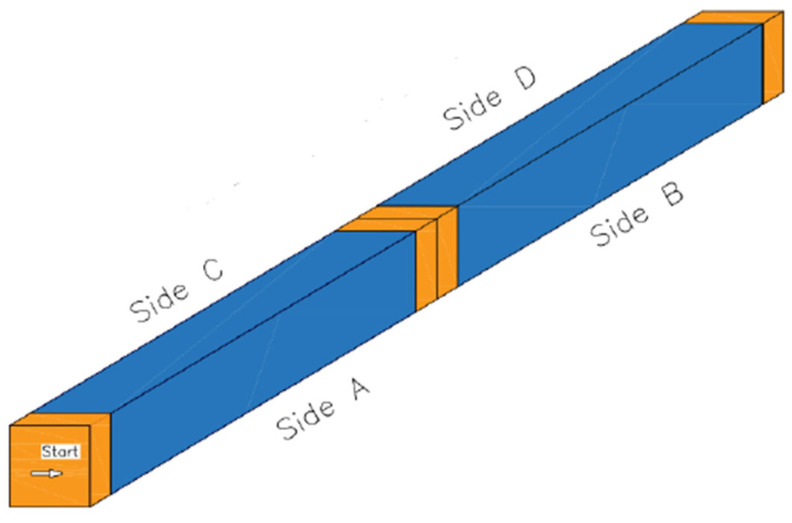
Schematic representation of the sandwich shear-face specimen.

Moreover, the buckles appear randomly on one of the four faces (A, B, C, or D) shown in Figure 18. Figure 18 depicts a diagram of the face parts of the sandwich specimen, specifically highlighting the shear side notation for the occurrence of shear wrinkling during a three-point bending test simulation. Table 4 summarizes the buckling load of the first eigenmode value and the location of wrinkling failure on the shear face of the specimen for different mesh sizes.

**Figure 19:**
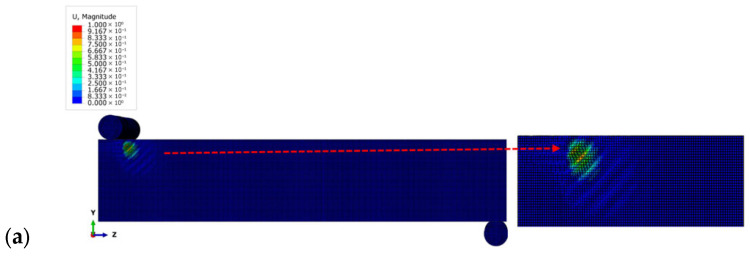

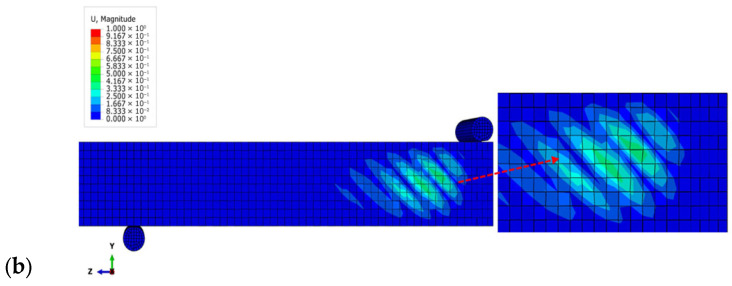
Wrinkling pattern for shear wrinkling case: 0.70 mm mesh size (**a**) and 5 mm mesh size (**b**).

Figure 19 shows the results of the numerical simulation study of shear wrinkling, with a particular focus on the shape of the wrinkles. These figures show how the wrinkling pattern changes as the mesh sizes vary. However, the buckle location and shape are inconsistent with those observed in experimental patterns, as they are always concentrated near the loading points. Furthermore, the position of the wrinkling observed in the experimental testing matches the shape and location of the wrinkling predicted using the numerical analysis, as illustrated in the previous subsection and in Figure 16. This consistent pattern further validates the accuracy of the numerical analysis in predicting the beam’s wrinkling behavior.

## 5. Tests/Modelling Comparisons

### 5.1. Compression Wrinkling

**Figure 20:**
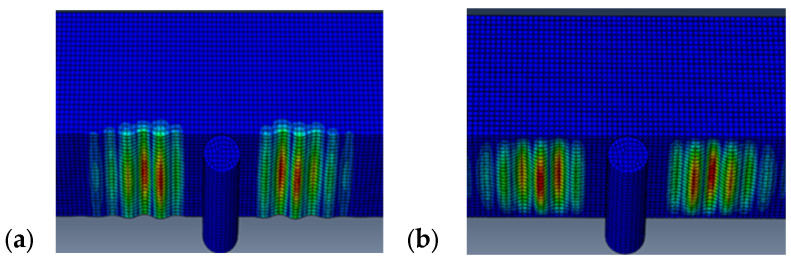
Finite element wrinkling pattern: (**a**) without shear face lamina and (**b**) with shear face lamina.

**Figure 21:**
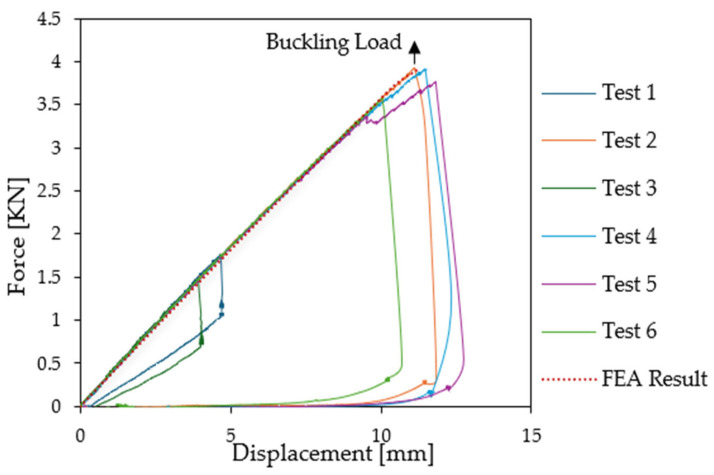
Load–displacement curve for compression wrinkling case: experimental and numerical results.

Both a linear buckling analysis and a simple geometrical nonlinear analysis without imperfection were conducted with a 1 mm mesh. The latter approach was used to accurately capture the wrinkling shape in the final step of the analysis (Figure 20) and to enable an accurate comparison of the load–displacement responses of the experiments and the numerical simulations (Figure 21). Two types of shell elements were tested for skins with and without shear stiffness (Kirchhoff or Mindlin elements), as shown in Figure 20. The overall wrinkling values (compression stress and full wavelength) are almost identical, but the shapes differ slightly since the Mindlin element better takes into accounts for the stiffness of the border effect and is closer to the experimental patterns. Figure 20 shows that the bending stiffness of the test specimens is well captured. The numerical analysis also predicts that the first eigenvalue buckling load for the converged mesh is 3.94 kN, whereas the experimental values given in Section 3.1 are as follows: 1.75 kN for Test 1; 3.91 kN for Test 2; 1.46 kN for Test 3; 3.91 kN for Test 4; 3.76 kN for Test 5; and 3.56 kN for Test 6. Despite the strong experimental dispersion, probably due to hand manufacturing and impregnation, as well as local imperfections, there is a strong correlation between the numerical analysis and the experimental tests. The results are also given in terms of face compression stresses in Table 5.

A simple calculation using the original Hoff and Mautner formula (C = 0.961, Equation (1)) also yields a close result of 332.45 MPa. However, both the numerical and analytical results overpredict the experimental results, which is probably due to the effect of initial imperfections caused by the manufacturing method. Furthermore, the position of the wrinkling observed in the experimental testing matches the shape and location of the wrinkling predicted using the numerical analysis, as illustrated in the previous subsection and in Figure 20. This consistent pattern further validates the accuracy of the numerical analysis in predicting the material’s wrinkling behavior.

### 5.2. Shear Wrinkling

**Figure 22:**
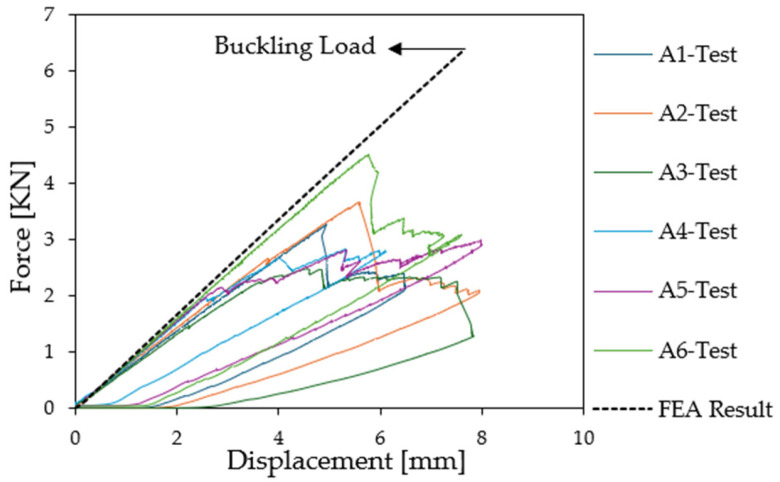
Load–displacement curve for the shear wrinkling case: experimental and numerical results.

In this case, only a linear buckling analysis is presented using a 1 mm mesh. Some nonlinear analyses with initial imperfections failed to capture the actual buckling loads. As demonstrated using the load displacement graph in Figure 22, the shear test modelling exhibits a significant correlation with the initial stiffness of the material properties. Conversely, the experimental results suggest that wrinkles form in the vicinity of the beam’s center, as shown in Figure 13, which was confirmed through numerical analysis of mesh convergence in Section 4.3. A first linear eigenvalue analysis predicted that the first buckling load would be 8.386 kN (Table 4, mesh size 0.7 mm); therefore, the wrinkling load was significantly overestimated.

This point must be analyzed in more depth in further studies by closely monitoring the initial imperfections and improving the manufacturing method. This can be achieved by replacing hand-impregnated plies with prepreg and using glue layers between the skin and layers to minimize the initial manufacturing imperfections, which are probably the cause of the experimental discrepancy.

## 6. Conclusions

This research provides a baseline for future investigations into shear and compression wrinkling in sandwich structures. The experimental specimen and methodology proposed in this paper were successful in creating compression and shear wrinkling. Numerical analysis showed that mesh size significantly impacts both the wrinkling load and the shape of the wrinkling pattern wavelength in sandwich composite structures. For compression failure, the numerical results correlate well with the experimental data, which indicates the reliability of the finite element models in these scenarios.

However, in shear failure tests, the proposed simple numerical analysis tends to significantly overestimate the wrinkling load and underestimate the wavelength. Furthermore, even when buckles are oriented at 45°, their location and size as determined by the numerical analysis differ significantly from the experimental results. Further investigations are required on this subject, both numerically and experimentally. It should be possible to use more advanced models incorporating initial imperfections, skin-interface delamination, and foam failure under tension or compression, as described in reference [41]. In general, shear wrinkling has not been widely studied in the literature, even though it can be a severe failure mode in lightweight aeronautical structures [5,38,41]. A more comprehensive analysis can therefore be made using this experimental device, with the use of prepregs, to monitor initial imperfections or, if possible, to create controlled initial imperfections, both in terms of shape and deflection.

## Figures and Tables

**Table 1:** Mechanical properties of skin materials.

Skin Material	E1 [MPa]	E2 [MPa]	Nu12	G12 [MPa]	G13 [MPa]	G23 [MPa]	Thickness, ts [mm]
Unidirectional	38,000	10,000	0.25	4000	4000	4000	0.30
Fabric	23,000	23,000	0.098	2900	2900	2900	0.16

**Table 2:** Mechanical properties of isotropic core material.

Core Material	E [MPa]	G [MPa]	Density [Kg/m^3^]	Shear Strength [MPa]	Thickness, tc [mm]
ROHACELL^®^ 51 WF	75	24	52	0.8	50

**Table 3:** Mechanical characteristics of plywood material (from [40]).

Plywood Material	E11 [MPa]	E22 [MPa]	E33 [MPa]	G12 [MPa]	G13 [MPa]	G23 [MPa]	Nu12 [MPa]	Nu13 [MPa]	Nu23 [MPa]	Density [Kg/m^3^]
Poplar	10,900	1003	469	818	752	120	0.392	0.318	0.329	480
Okoumé	9000	828	387	675	621	99	0.392	0.312	0.329	430

**Table 4:** Summary of wrinkling load, wavelength, and location of wrinkling failure.

Location of Wrinkling Failure	Full Wave Length [mm]	Wrinkling Load [kN]	Mesh Size [mm]
Side D	3.97	8.386	0.7
Side A	4.2	9.247	0.75
Side B	4.45	9.41	1
Side B	5.66	10.83	2
Side B	8.42	11.71	3
Side B	8.88	13.48	4
Side B	11.2	15.34	5

**Table 5:** Summary of analysis results for FEA and experimental analysis for compression wrinkling.

Full Wavelength [mm]	Wrinkling Stress [MPa]	References	
12.75	343.74	With shear face lamina	**FEA**
12.75	342.64	Without shear face lamina	
		Test 1	**Experimental**
18	313	Test 2	
		Test 3	
9.8	264	Test 4	
10.2	254	Test 5	
18	270	Test 6	

## Data Availability

The original contributions presented in this study are included in the article. Further inquiries can be directed to the corresponding author.

## References

[1] Zenkerts D. (1997). The Handbook of Sandwich Construction.

[2] Ley R.P., Lin W., Mbanefo U. (1999). Facesheet Wrinkling in Sandwich Structures.

[3] Palomba G., Epasto G., Crupi V. (2022). Lightweight sandwich structures for marine applications: A review. Mech. Adv. Mater. Struct..

[4] Manalo A., Aravinthan T., Fam A., Benmokrane B. (2017). State-of-the-Art review on FRP sandwich systems for lightweight civil infrastructure. J. Compos. Constr..

[5] Castanié B., Bouvet C., Ginot M. (2020). Review of composite sandwich structure in aeronautic applications. Compos. Part C.

[6] Ginot M., D’Ottavio M., Polit O., Bouvet C., Castanié B. (2021). Benchmark of wrinkling formulae and methods for pre-sizing of aircraft lightweight sandwich structures. Compos. Struct..

[7] Daniel I.M., Gdoutos E.E., Wang K.A., Abot J.L. (2002). Failure modes of composite sandwich beams. Int. J. Damage Mech..

[8] Gdoutos E.E., Daniel I.M., Wang K.A., Mccormick R.R. (2003). Compression facing wrinkling of composite sandwich structures. Mech. Mater..

[9] Léotoing L., Drapier S., Vautrin A. (2002). First applications of a novel unified model for global and local buckling of sandwich columns. Eur. J. Mech. A Solids.

[10] Léotoing L., Drapier S., Vautrin A. (2002). Nonlinear interaction of geometrical and material properties in sandwich beam instabilities. Int. J. Sol. Struct..

[11] D’Ottavio M., Polit O., Ji W., Waas A.M. (2016). Benchmark solutions and assessment of variable kinematics models for global and local buckling of sandwich struts. Compos. Struct..

[12] Di Cara G., D’Ottavio M., Polit O. (2024). Variable kinematics finite plate elements for the buckling analysis of sandwich composite panels. Compos. Struct..

[13] Zhang Y., Cao P., Yang X., Niu K. (2025). Buckling modeling and numerical validation of composite panels for sandwich structures. J. Sand. Struct. Mater..

[14] Lopatin A.V., Morozov E.V. (2008). Symmetrical Facing Wrinkling of Composite Sandwich Panels. J. Sand. Struct. Mater..

[15] Benson A.S., Mayers J. (1967). General Instability and Face Wrinkling of Sandwich Plates—Unified Theory and Applications. AIAA J..

[16] Wei X., Wu Q., Gao Y., Yang Q., Xiong J. (2022). Composite honeycomb sandwich columns under in-plane compression: Optimal geometrical design and three-dimensional failure mechanism maps. Eur. J. Mech. A Solids.

[17] Hoff N.J., Mautner S.E. (1945). The Buckling of Sandwich-Type Panels. J. Aeronaut. Sci..

[18] Yusuff S. (1955). Theory of Wrinkling in Sandwich Construction. J. R. Aeronaut. Soc..

[19] Allen H.G. (1969). Wrinkling and other forms of local instability. Analysis and Design of Structural Sandwich Panels.

[20] Daniel I.M., Abot J.L. (2000). Fabrication, testing and analysis of composite sandwich beams. Comp. Sci. Technol..

[21] Fagerberg L., Zenkert D. (2005). Imperfection-induced wrinkling material failure in sandwich panels. J. Sand. Struct. Mater..

[22] Fagerberg L., Zenkert D. (2005). Effects of anisotropy and multiaxial loading on the wrinkling of sandwich panels. J. Sand. Struct. Mater..

[23] Manshadi B.D., Vassilopoulos A.P., de Castro J., Keller T. (2012). Modeling of Buckling and Wrinkling Behavior in GFRP Plate and Sandwiches Subjected to Biaxial Compression–Tension Loading. J. Compos. Const..

[24] Khalili S.M.R., Kheirikhah M.M., Malekzadeh Fard K. (2014). Biaxial wrinkling analysis of composite-faced sandwich plates with soft core using improved high-order theory. Eur. J. Mech. A Solids.

[25] Birman V., Costa H. (2017). Wrinkling of Functionally Graded Sandwich Structures Subject to Biaxial and In-Plane Shear Loads. ASME. J. Appl. Mech..

[26] Birman V., Bert C.W. (2004). Wrinkling of composite-facing sandwich panels under biaxial loading. J. Sand. Struct. Mater..

[27] Manshadi B.D., Vassilopoulos A.P., de Castro J., Keller T., Ye L., Feng P., Yue Q. (2011). Shear Wrinkling of GFRP Webs in Cell-Core Sandwiches. Advances in FRP Composites in Civil Engineering.

[28] Manshadi B.D., Vassilopoulos A.P., de Castro J., Keller T. (2012). Instability of thin-walled GFRP webs in cell-core sandwiches under combined bending and shear loads. Thin-Walled Struct..

[29] Rodríguez-Ramírez J.D.D., Castanié B., Bouvet C. (2020). Damage Mechanics Modelling of the shear nonlinear behavior of Nomex honeycomb core Application to sandwich beams. Mech. Adv. Mater. Struct..

[30] Chen J., Hao N., Song Y., Yang J., He C. (2022). Shear properties of 3D-printed grid beetle elytron plates. J. Mater. Sci..

[31] Tuo W., Yan L., Chen J., Chang X., Gao Y., Wang Y. (2020). Effect of the length of basalt fibers on the shear mechanical properties of the core structure of biomimetic fully integrated honeycomb plates. J. Sandw. Struct. Mater..

[32] Kassapoglou C. (2010). Design and Analysis of Composite Structures (with Application to Aerospace Structures).

[33] Wagner H., Ballerstedt W. (1935). Tension Field in Originally Curved, Thin Sheets During Shearing Stresses.

[34] Barrau J.J., Crézé S., Castanié B. (2005). Buckling and post-buckling of beams with flat webs. Thin-Walled Struct..

[35] Youngquist W.G., Kuenzi E.W. (1961). Shear and Torsion Testing of Wood, Plywood, and Sandwich Constructions at the U. S. Forest Products Laboratory. Symposium on Shear and Torsion Testing. ASTM Selected Technical Papers.

[36] Farley G.L., Baker D.J. (1983). In-plane shear test of thin panels. Exp. Mech..

[37] Bertolini J., Castanié B., Barrau J.-J., Navarro J.-P., Petiot C. (2009). Multi-level experimental and numerical analysis of composite stiffener debonding. Part 2: Element and panel level. Compos. Struct..

[38] Ginot M., Bouvet C., Castanié B., Serra J., Mahuet N. (2023). Local buckling on large sandwich panels used in light aviation: Experimental setup and failure scenarios. Compos. Struct..

[39] Castanié B., Passieux J.C., Périé J.N., Bouvet C., Dufour J.E., Serra J. (2024). Multiaxial loading of aeronautic composite structures at intermediate scale: A review of VERTEX developments. Comp. Part C..

[40] Susainathan J., Eyma F., De Luycker E., Cantarel A., Castanie B. (2020). Numerical modeling of impact on wood-based sandwich structures. Mech. Adv. Mater. Struct..

[41] Ginot M., Bouvet C., Castanié B., D’Ottavio M., Serra J., Mahuet N. (2023). Local buckling on large sandwich panels applied to light aviation: Experimental and computation dialogue. Int. J. Solids Struct..

